# Supplementary figures and images for: Practice walking on a treadmill-mounted balance beam modifies beam walking sacral movement and alters performance in other balance tasks
Source: PLoS One. 2023 Jun 15;18(6):e0283310. doi: 10.1371/journal.pone.0283310 (PMC10270570; doi:10.1371/journal.pone.0283310)

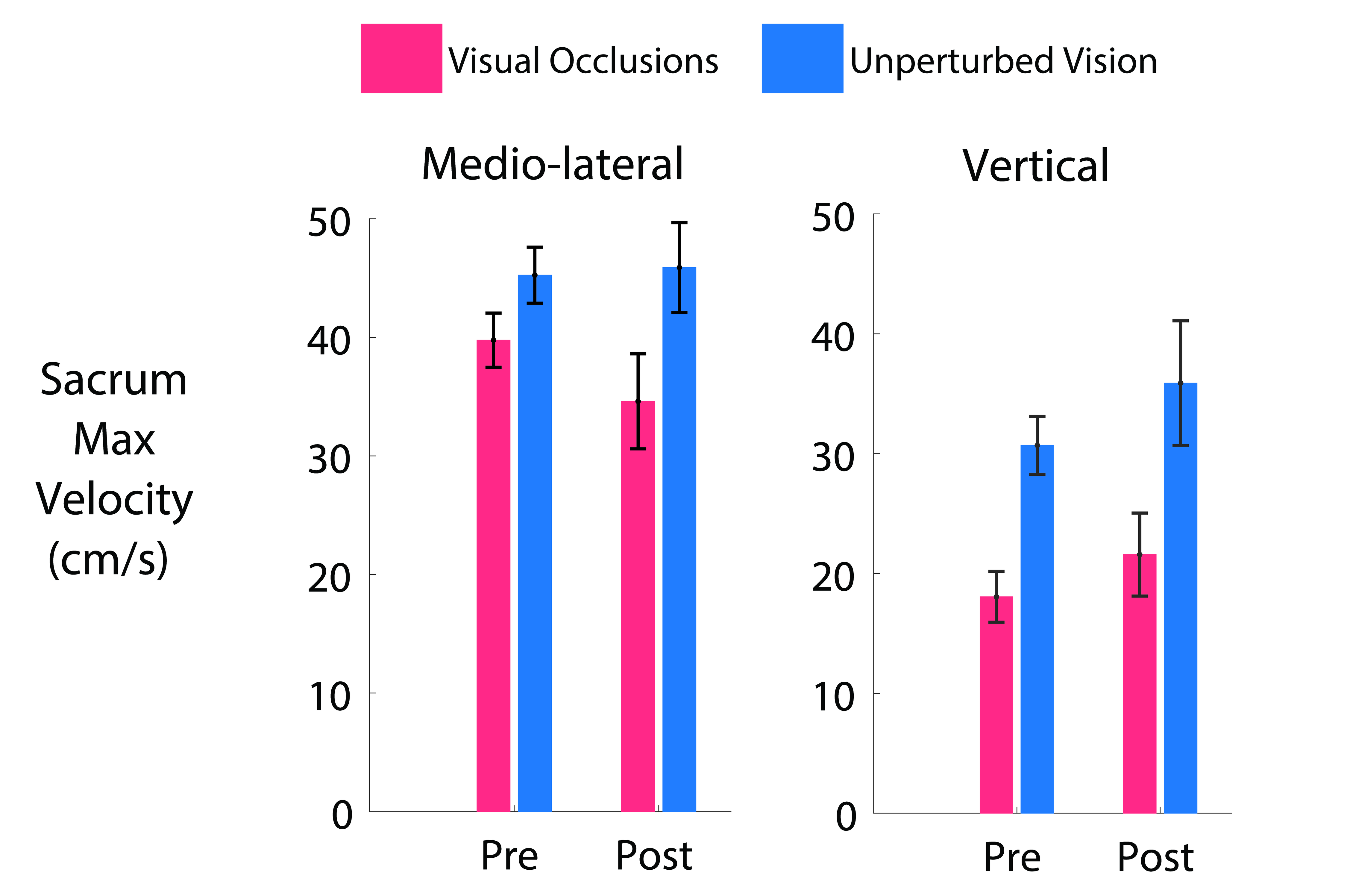

Supplement: S1 Fig — Medio-lateral and vertical sacral marker maximum velocity for the visual occlusions (red) and unperturbed vision group (blue) before (Pre) and after (Post) the training. In the medio-lateral direction there was a significant main effect of training type (F(1,16) = 5.1, p = .04, partial η2 = .24). The visual occlusions group also showed a decline in maximum sacrum velocity between pre- and post-test. In the vertical direction we saw a main effect of training type (F(1,16) = 8.7, p = .009, partial η2 = .35) and a main effect of test-trial (F(1,16) = 4.9, p = .04, partial η2 = .24). Error bars represent standard error of the mean. (TIF) [file pone.0283310.s001.tif]

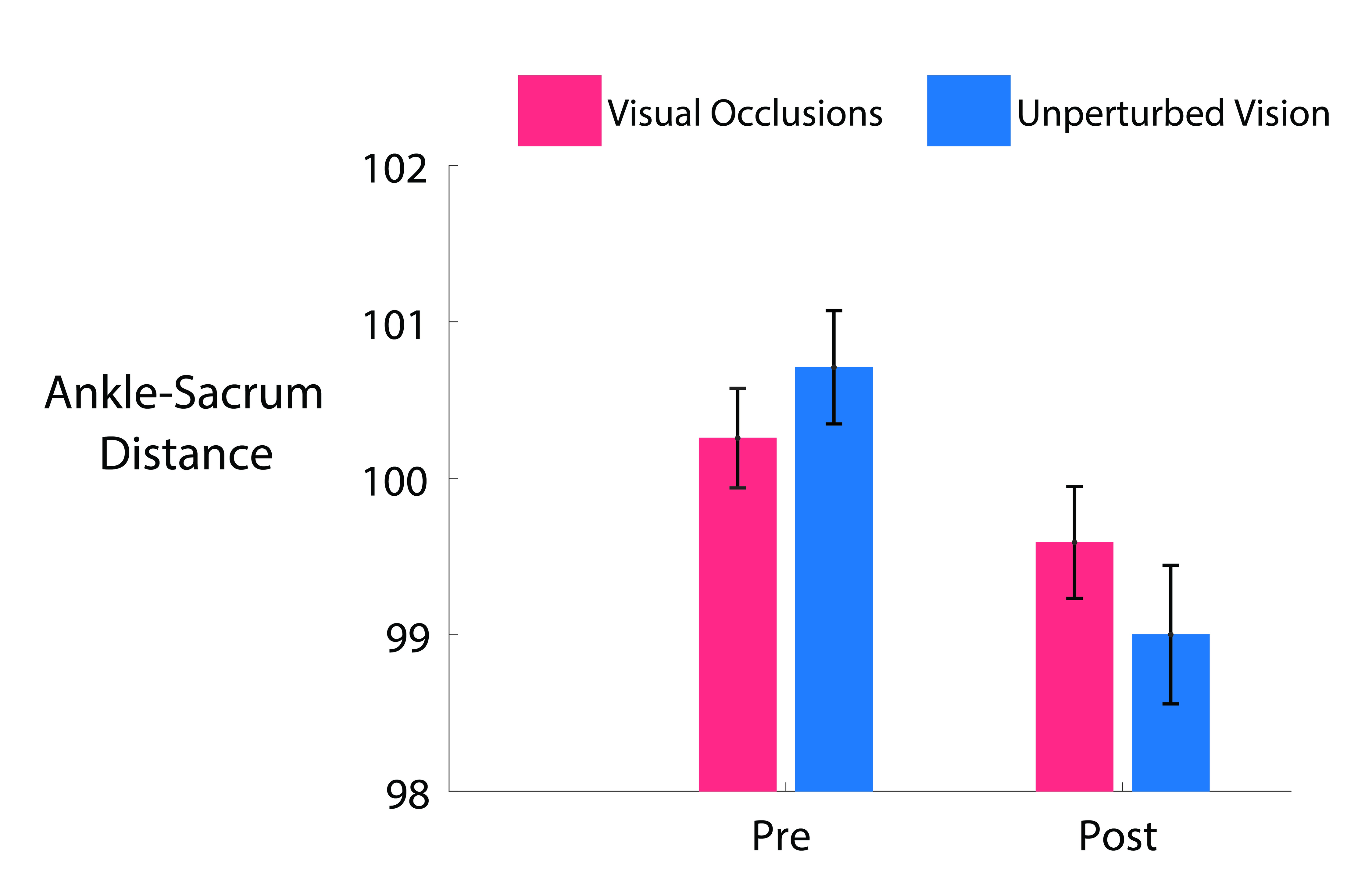

Supplement: S2 Fig — Normalized ankle-sacrum distance for the visual occlusions (red) and unperturbed vision group (blue) before (Pre) and after (Post) the training. Both groups showed a significant reduction of ankle-sacrum distance between pre- and post-test (F(1,16) = 15.8, p = .001, partial η2 = .5), meaning both adapted a more crouched position after training. The visual occlusions group showed ~0.7 cm reduction, while the unperturbed vision group showed a larger deduction of ~1.7 cm. Error bars represent standard error of the mean. (TIF) [file pone.0283310.s002.tif]

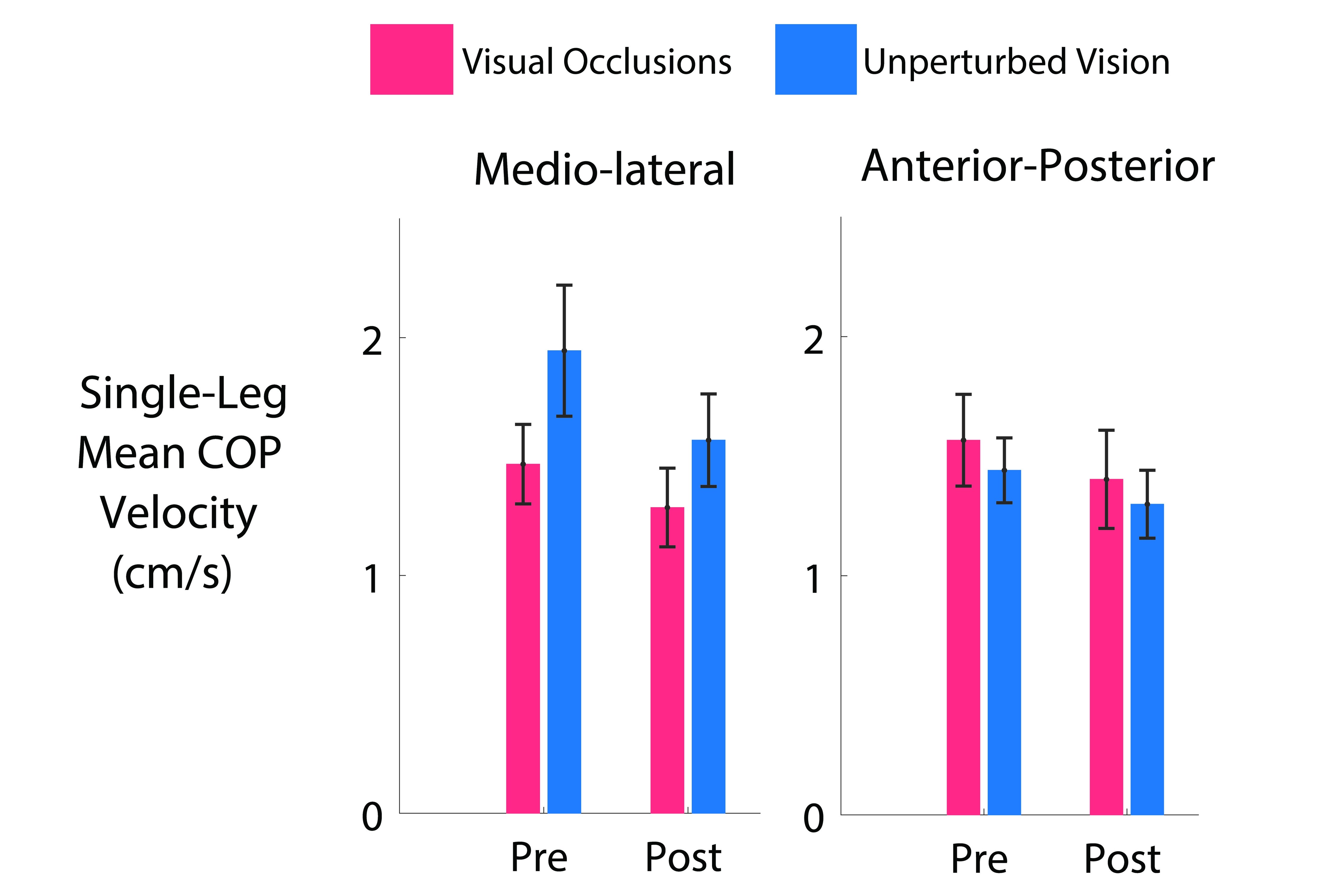

Supplement: S3 Fig — Mean mediolateral and anterior-posterior center of pressure (CoP) velocity during the single-leg stance task. Both groups showed a decrease in CoP velocity in the medio-lateral and anterior-posterior direction. There was a significant main effect of test-trial in the medio-lateral (F(1, 14) = 14, p = .002, partial η2 = .50) and anterior-posterior direction (F (1, 14) = 8.8, p = .01, partial η2 = .38). Error bars represent standard error of the mean. (TIF) [file pone.0283310.s003.tif]
